# The neurovascular unit as a selective barrier to polymorphonuclear granulocyte (PMN) infiltration into the brain after ischemic injury

**DOI:** 10.1007/s00401-012-1076-3

**Published:** 2012-12-27

**Authors:** Gaby Enzmann, Caroline Mysiorek, Roser Gorina, Yu-Jung Cheng, Sharang Ghavampour, Melanie-Jane Hannocks, Vincent Prinz, Ulrich Dirnagl, Matthias Endres, Marco Prinz, Rudi Beschorner, Patrick N. Harter, Michel Mittelbronn, Britta Engelhardt, Lydia Sorokin

**Affiliations:** 1 Theodor Kocher Institute, University of Bern, Freiestrasse 1, 3012 Bern, Switzerland; 2 Institute of Physiological Chemistry and Pathobiochemistry, University of Münster, Waldeyerstrasse 15, 48149 Münster, Germany; 3 Department of Neurology, Berlin, Germany; 4 Center for Stroke Research Berlin, Charité University, Berlin, Germany; 5 Department of Neuropathology, University of Freiburg, Freiburg, Germany; 6 BIOSS Centre for Biological Signaling Studies, University of Freiburg, Freiburg, Germany; 7 Department of Neuropathology, Institute of Pathology and Neuropathology, University of Tübingen, Tübingen, Germany; 8 Institute of Neurology (Edinger Institute), University of Frankfurt, Frankfurt, Germany

**Keywords:** Neurovascular unit, Polymorphonuclear granulocyte, Migration, Human, Mouse

## Abstract

The migration of polymorphonuclear granulocytes (PMN) into the brain parenchyma and release of their abundant proteases are considered the main causes of neuronal cell death and reperfusion injury following ischemia. Yet, therapies targeting PMN egress have been largely ineffective. To address this discrepancy we investigated the temporo-spatial localization of PMNs early after transient ischemia in a murine transient middle cerebral artery occlusion (tMCAO) model and human stroke specimens. Using specific markers that distinguish PMN (Ly6G) from monocytes/macrophages (Ly6C) and that define the cellular and basement membrane boundaries of the neurovascular unit (NVU), histology and confocal microscopy revealed that virtually no PMNs entered the infarcted CNS parenchyma. Regardless of tMCAO duration, PMNs were mainly restricted to luminal surfaces or perivascular spaces of cerebral vessels. Vascular PMN accumulation showed no spatial correlation with increased vessel permeability, enhanced expression of endothelial cell adhesion molecules, platelet aggregation or release of neutrophil extracellular traps. Live cell imaging studies confirmed that oxygen and glucose deprivation followed by reoxygenation fail to induce PMN migration across a brain endothelial monolayer under flow conditions in vitro. The absence of PMN infiltration in infarcted brain tissues was corroborated in 25 human stroke specimens collected at early time points after infarction. Our observations identify the NVU rather than the brain parenchyma as the site of PMN action after CNS ischemia and suggest reappraisal of targets for therapies to reduce reperfusion injury after stroke.

**Electronic supplementary material:**

The online version of this article (doi:10.1007/s00401-012-1076-3) contains supplementary material, which is available to authorized users.

## Introduction

Reperfusion of cerebral vessels after transient occlusion is associated with immune cell recruitment, which contributes to both damage of the vessel and the surrounding tissue. Polymorphonuclear granulocytes (PMNs) are considered to play a prominent role in microvascular responses to ischemia via protease-mediated tissue damage and neuronal cell death during reperfusion by direct contact with CNS tissue [2, 7, 19, 39, 49, 60]. This necessitates PMN extravasation across the blood–brain barrier (BBB) into the brain parenchyma at early stages after the ischemic insult, prior to terminal neuronal damage [25].

Yet, the effects of experimental neutropenia in rodent stroke models range from decreased infarct volume [12, 62, 75] to little or no influence on lesion size [11, 44, 45], and PMN mobilization from the bone marrow induced by granulocyte colony stimulating factor (G-CSF) does not worsen clinical outcome in murine stroke models [83] or in a recent clinical phase II trial (AXIS) [76]. Furthermore, although some animal experiments targeting adhesion molecules mediating the multi-step PMN migration across inflamed microvessels have shown reduced infarct size [25], clinical trials targeting PMN adhesion to endothelial ICAM-1 [9, 36, 47] or aiming at preventing PMN infiltration into the brain by neutralizing the αMβ2-integrin (CD11b/CD18) [54] have failed to alleviate stroke severity, suggesting that the mode of PMN action requires reassessment.

These discrepancies may reflect differences between the pathogenic mechanisms involved in ischemia/reperfusion in animal experiments and human stroke cases or, alternatively, could be linked to the assumption that PMNs use ICAM-1 and β2-integrins to migrate into the brain parenchyma in reperfusion injury, as in other inflammatory scenarios. This, however, has not been formally shown and most PMN depletion studies or adhesion molecule blocking studies after cerebral ischemia either did not assess PMN/immune cell infiltration [15, 16, 67] or employed methods that do not permit unequivocal identification of PMNs [37, 52, 61, 78]. A common problem has been the use of broad specificity reagents such as the antibody clone, RB6-8C5, targeting the Gr-1 antigen, which recognizes both Ly6G and Ly6C and therefore stains PMNs and monocytes [35]. Similarly, myeloperoxidase staining identifies PMNs but also monocytes and activated microglial cells [13]. Hence, the use of such broad specificity reagents in flow cytometry and/or immunohistochemistry inevitably results in an over-estimation of PMN numbers. It is therefore important to reassess the role of PMNs after cerebral ischemia using more specific immune cell markers that are now available, in particular in relation to their association with adhesion molecules and their precise localization within the vasculature and/or brain parenchyma.

That CNS vessels and neurons are functionally coupled is well illustrated by the rapid response of neurons to focal ischemia. Mechanistically, this is achieved by the neurovascular unit (NVU), composed of a monolayer of specialized endothelial cells (EC) interconnected by complex tight junctions, the underlying endothelial basement membrane (BM) and a second BM, known as the parenchymal BM as it marks the border to the CNS parenchyma. Together with the associated ensheathing layer of astrocyte endfeet, the parenchymal BM defines the glia limitans [3, 64, 89], which is structurally and functionally interconnected to the surrounding neurons via astrocytes and microglia [14, 18]. With the exception of capillaries, where endothelial and parenchymal BMs fuse to form one composite BM, in all brain parenchymal vessels the endothelial and parenchymal BMs are structurally and biochemically distinct entities, which define the inner and outer limits of the perivascular space.

The NVU strictly controls immune cell emigration from the blood vessel that requires a cascade of adhesive interactions that are well described for neuroinflammation [32]. Due to the specialized structure of the NVU, leukocyte entry into the brain parenchyma involves two differently regulated steps: migration of leukocytes across the endothelium into the perivascular space and progression across the glia limitans into the brain parenchyma. In a murine model of multiple sclerosis, experimental autoimmune encephalomyelitis (EAE), induction of disease symptoms occurs only upon immune cell penetration of the glia limitans into the CNS parenchyma, whereas accumulation of inflammatory cells within the perivascular space does not translate into clinical disease [1, 8, 88], highlighting the significance of the glia limitans as the effective border to the CNS parenchyma.

While histological analyses of brain samples from murine models of transient ischemia and human stroke tissues have detected “inflammatory infiltrates” in the brain [33, 68], there has been little attempt to either specifically identify PMNs using unique molecular markers or to localize their precise position within the brain at defined time points after ischemic stroke. This study presents a collaborative investigation involving stroke researchers, neuropathologists, and basic scientists to determine the temporo-spatial relationship between immune cells and blood vessel micro-architecture in the mouse and human brain at early (acute) stages after ischemia using a panel of markers for different subsets of myeloid cells, endothelial cell adhesion molecules, and the BMs of the NVU. We employ the specific PMN marker, Ly6G [17], in immunohistochemistry and in double and triple immunofluorescence confocal microscopy to precisely localize PMNs in relation to the cellular and BM components of the NVU, and to investigate correlations between PMN localization and altered vessel permeability or expression of endothelial cell adhesion molecules known to be involved in PMN rolling (P-selectin), arrest and crawling (ICAM-1, ICAM-2) or diapedesis (PECAM-1, CD99, JAM-A). An in vitro model for the BBB [82], where PMN migration across an endothelial monolayer is measured under physiological flow, is employed to investigate the effects of transient oxygen–glucose deprivation followed by reoxygenation. Our mouse and human data highlight the need for a critical reappraisal of the precise site of PMN action after stroke and molecular targets for therapies to reduce reperfusion injury after stroke.

## Materials and methods

### Animals

151 male C57BL/6 and 129Sv 8–12-week-old mice were employed (Table 1). Animal experiments were performed according to Swiss (56/08) and German (G0383/09) legislation.

**Table 1:** Mouse tissues analyzed

Mouse strain	Ischemia (min)	Acute reperfusion	Surgery performed	Total animals analyzed	Animals analyzed by IH	Antibodies employed^c^	Animals analyzed by IF	Antibodies employed^c^
C57Bl/6	30	6, 12, 18, 24, 48, 72 h and 2 weeks	Berlin, Bern	59	12	Leukocyte markers: CD45, Gr-1, Ly6G, F4/80, CD11b, Endothelial cell markers: PECAM-1, VCAM-1, ICAM-1, ICAM-2, P-selectin	47^a^	BM: Pan-laminin, laminin α5, laminin α2, collagen IV Leukocyte markers: CD45, Ly6G, Ly6C, F4/80, CD11b, CD41 Endothelial cell markers: PECAM-1, VCAM-1, ICAM-1, ICAM-2, P-selectin.
C57Bl/6	60	6, 12, 18, 24, 48, 72 h and 2 weeks	Berlin, Bern	68	8		60^a^	
C57Bl/6	90	3, 18, 48 h	Berlin	9	3		6^b^	
129 Sv	30	6, 72 h	Berlin	6	3		3	
129 Sv	90	3, 18, 48 h	Berlin	9	3		6	

^a^18, 24, 48, 72 h and 2 week samples analyzed

^b^18 h, 48 h analyzed

^c^See Table 2

### Transient middle cerebral artery occlusion (tMCAO)

Transient focal ischemia using the intraluminal filament model was performed [26]. The left middle cerebral artery was unilaterally occluded for 30, 60, and 90 min, after which the filament was withdrawn and the tissue was reperfused for varying lengths of time, resulting in the following ischemia/reperfusion (I/R) protocols: I/R: 30 min/6, 12, 18, 24, 48, 72 h, 1 and 2 weeks; 60 min/6, 12, 18, 24, 48, 72 h, 1 and 2 weeks; 90 min/3, 18, 48 h). Motor–sensory scores of the mice analyzing gait disturbances were determined [10, 27] prior to termination of the experiments. To account for experimenter variability, tMCAO was performed in two independent laboratories and brains were distributed to two separate laboratories for independent immunohistological and immunofluorescence analyses. All analyses included coronal sections through the core of the lesion and adjacent penumbra (Bregma 0.50 mm), and caudal to the ischemic area (Bregma −2.46 mm). Hence, all analyses were standardized to the Stroke Therapy Academic Industry Roundtable (STAIR) criteria [34].

### Flow cytometry

Inflammatory cells from ischemic ipsilateral and contralateral brain hemispheres were isolated and stained as described previously [28, 30]. Briefly, 6 anaesthetized stroke mice in 3 independent experiments were perfused with 297.4 mosm/l phosphate buffered saline (PBS), pH 7.4, to remove peripheral blood, their brains dissected, and ischemic and contralateral cerebral hemispheres separated. The tissue was then mechanically dissociated, digested with collagenase VIII and DNAse I, filtered, and cells were separated by centrifugation through a Percoll-gradient. CD45^high^ inflammatory cells and CD45^intermediate^ microglia were collected from the interphase and stained for Ly6G to identify PMNs. Flow cytometry was performed using a FACSCalibur; CellQuest (Becton–Dickinson) and FlowJo (Tree Star Inc.) software were employed for data analysis.

### Immunohistochemistry

Mice were perfused with 1 % paraformaldehyde (PFA) in PBS, pH 7.4, their brains removed, embedded in TissueTek (OCT Compound, Haslab) and frozen in a dry ice/isopentane bath. Cryostat sections (6 μm) spanning the whole lesion area were prepared, fixed in −20 °C acetone and stained using a three-step immunoperoxidase staining kit (Vectastain). Primary antibodies employed are listed in Table 2. Secondary antibodies included biotinylated anti-rat and goat antibodies and were consecutively incubated with avidin–biotin complex (ABC) and peroxidase substrate solution (AEC, Vectastain). Sections were assessed using a Nikon Eclipse E600 microscope equipped with a digital camera.

**Table 2:** Primary antibodies to extracellular matrix molecules and inflammatory cells

Molecule	Antibody name/clone	Marker	IF/IH^a^	Reference/source
Mouse				
Pan-Laminin	455	All BMs	IF, IH	[80]^b^
Laminin α2	401	Parenchymal BM	IF	[77]^b^
Laminin α4	377	Endothelial BM	IF	[73]^b^
Laminin α5	405	Endothelial BM	IF	[80]^b^
Collagen IV	–	All BMs	IF	[86]^c^
P-Selectin	Rabbit anti-P-selectin	Activated endothelium	IH	[22], BD Pharmingen
ICAM-1	25ZC7	Endothelial adhesion molecule	IF, IH	[72], BD Pharmingen
ICAM-2	3C4	Endothelial adhesion molecule	IH	BD Pharmingen
VCAM-1	9DB3	Endothelial adhesion molecule	IF, IH	[29], BD Pharmingen
PECAM-1	Mec13.3	Endothelial junctions	IH	BD Pharmingen
Ly6G	1A8	PMNs	IF, IH	[17], BD Pharmingen
Ly6C	AL-21	Monocytes	IF	[17], BD Pharmingen
Gr-1	RB6-8C5	PMNs, monocytes	IF, IH	[35], BD Pharmingen
F4/80	A3-1	Macrophages	IF, IH	BD Pharmingen
CD45	30G12 M1-9	All leukocytes	IF IH	BD Pharmingen
CD11b/Mac-1	M1/70	Microglia, monocytes, macrophages	IF, IH	BD Pharmingen
CD41	MWreg30	Platelets	IF, IH	BD Pharmingen
IgG	236 BA-9200	Murine IgG	IF, IH	BD Pharmingen

Antigen	Antibody name/clone	Marker	IF/IH^a^	Reference/source
Human				
CD3	Polyclonal	T lymphocytes	IH	Dako
CD15	C3D-1	PMNs, monocytes	IH, IF	Dako
CD45	2B11	All leukocytes	IH, IF	Dako
CD68	PG-M1	Activated macrophages, monocytes, microglia	IH, IF	Dako
LCA	PD7/26	All leukocytes	IH	Dako
Hif1alpha	NB 100-134	Hypoxia	IH	Novus biologicals
c.Caspase-3	Asp 175	Apoptotic cells	IH	Cell signaling

^a^Employed in *IF* immunofluorescence or *IH* immunohistochemistry

^b^Provided by L. Sorokin

^c^Kindly provided by K. von der Mark (Erlangen)

### Immunofluorescence

Immunofluorescence staining was performed on non-perfused, snap frozen tissues. 5-μm cryostat sections were fixed in −20 °C methanol. For confocal microscopy or 3D reconstructions, tissues were fixed in 1.5 % PFA in PBS, pH 7.4, for 1.5 h at 4 °C, embedded in 1 % agarose in PBS, pH 7.4, and 100-μm sections were prepared using a Zeiss vibratome. Thick and thin sections were treated with PBS, pH 7.4, plus 1 % BSA before incubation at 4 °C with primary antibody (Table 2). Secondary antibodies included goat anti-rabbit and donkey anti-rat IgG conjugated with Alexa Fluor 488 or Alexa Fluor 594 (Molecular Probes). Sections were examined using a Zeiss AxioImager microscope equipped with epifluorescent optics and documented using a Hamamatsu ORCA ER camera or with a Zeiss confocal laser scanning system LSM 510meta. Images were analyzed using Volocity 5.2 software (ImproVision, Perkin Elmer).

To quantitate PMN numbers within vessels, in the perivascular space or in the brain parenchyma, 100-μm coronal sections throughout the entire brain were double immunofluorescently stained for pan-laminin and Ly6G or CD45. Ly6G^+^ or CD45^+^ cells located within or outside of the pan-laminin staining were counted in optical sections throughout the thick sections and expressed per 0.001 mm^3^ brain volume, and normalized to the proportion of the brain volume occupied by vessels (i.e., total cells × vessel volume/total brain volume). This calculation reflects the relative numbers of PMNs accumulated in the different brain areas. Cells in or associated with vessels were similarly expressed per m^3^ brain volume, and subsequently normalized to the proportion of the brain volume occupied by vessels. The cells in or associated with vessels as a percentage of the total cell number were calculated from these values. At least 5 fields of view were analyzed/section, and at least 3 different thick sections were analyzed within ischemic lesions from 2 to 3 mice. Statistical analyses were employed to determine whether PMN numbers were significantly higher in meninges, cortex or striatum (one-way ANOVA), and to test for deviations from 100 % Ly6G^+^ cells in or in association with vessels (one-way ANOVA).

### In vivo blood–brain barrier permeability

tMCAO/24 h reperfusion mice were injected with 2 % Hoechst 33258 (Calbiochem) plus 2 % Evans Blue (Alfa Aesar) (total of 2 mice), or with a combination of 3 kDa Texas Red-conjugated Dextran and 10 kDa FITC-conjugated Dextran, 100 μg each (Molecular Probes) (total of 2 mice). The dyes were allowed to circulate for 30 min and 15 min, respectively, before sacrificing the mice. Only dextran-injected mice were perfused with PBS, pH 7.4, followed by 4 % formaldehyde/PBS, pH 7.4, and brains were frozen. Brains from Hoechst/Evans blue-injected mice were snap frozen in TissueTek in a dry ice/isopentane bath. Cryosections were analyzed for possible extravasation of tracers from microvessels of the stroke-afflicted hemisphere, with extravasation across the fenestrated endothelium of the choroid plexus serving as internal positive control.

### In vitro blood–brain barrier model

Mixed glial cell cultures enriched in astrocytes [41] were cocultured with primary mouse brain microvascular endothelial cells (pMBMECs) [82] and subjected to oxygen and glucose deprivation (OGD) by adding glucose- and serum-free medium and keeping the cells under anoxic conditions using GasPack EZ bags (Becton–Dickinson) for 4 h. For normoxic controls, cells were exposed to serum-free DMEM containing glucose during the OGD period. 4 h IL-1β (20 ng/ml) stimulated pMBMECs under normoxic conditions were also employed. After 20 h of reoxygenation, the endothelial monolayer, grown on an insert (Millicell CM, Millipore), was placed on a flow chamber and highly purified bone marrow derived PMNs were perfused over the pMBMECs. PMNs were allowed to accumulate for 4 min at low shear stress (0.25 dyn/cm^2^); the subsequent PMN interaction with pMBMECs under physiological shear stress (1.5 dyn/cm^2^) was recorded with an inverted microscope (AxioObserver Z1, Carl Zeiss) at 20× magnification (objective EC Plan Neofluar × 10/0.3). Time-lapse videos were created by taking one image every 20 s over a 20-min period (AxioVision, Carl Zeiss). *Image J* software was used for the analysis of the movies. pMBMECs were also immunostained for ICAM-1 and ICAM-2 and counter-stained with Hoechst dye to show the cell nuclei.

### Human samples

Brain autopsy and biopsy material of twenty-five stroke patients (Supplementary Table 1) was analyzed by H&E, chloracetate esterase, myeloperoxidase, immunohistochemical, or immunofluorescent (Table 2) staining in accordance with the local ethics committee. The DNA-binding dye TO-PRO-3 (Invitrogen, Germany) was employed to mark all nuclei. Infarct staging was performed according to the 3-stage stroke classification frequently used in neuropathological diagnostics, which was first described in detail by Hugo Spatz in 1939 [81], and of which cellular reactions have been more sophisticatedly deciphered over the years (for review see [24]). Histopathologically, the infarct lesions were classified into stage I (acute), stage II (subacute), and stage III (chronic). Human brains were fixed in 4 % phosphate-buffered formaldehyde; pH 7.4, embedded in paraffin, and 3-μm sections were analyzed. Immunohistochemical single and double stainings were performed using the Benchmark and DiscoveryXT immunohistochemistry systems (Ventana/Roche, France) and counterstained with hematoxylin. Primary antibodies employed are listed in Table 2. Immunofluorescence images were analyzed and recorded using a Leica TCS SP confocal microscope, and the EZ-C1 software. After recording, digital images were further processed and adjusted for brightness, contrast, and colour balance with ImageJ (NIH).

## Results

Since C57BL/6 mice develop larger infarcts after tMCAO than Sv129 mice [59, 66] and Sv129 mice exhibit higher levels of circulating PMNs compared with C57BL/6 mice [65], we studied tMCAO in both mouse strains in parallel (Table 1).

### Temporal and spatial appearance of polymorphonuclear granulocytes (PMNs) in the brain after transient middle cerebral artery occlusion (tMCAO)

To assess effects of ischemia duration on immune cell recruitment, C57BL/6 mice were subjected to tMCAO for 30, 60, and 90 min, and Sv129 mice to 30 and 90 min (Table 1). Since the lesion matures over time, reperfusion times of 6, 12, 18, 24, 48, 72 h, 1 and 2 weeks were examined to cover the entire acute phase of reperfusion injury in all tMCAO scenarios (Table 1). Tissue damage caused by these tMCAO times is well characterized, with 30 min causing mainly selective nerve cell injury and astrogliosis in the striatum, and 60 and 90 min causing selective nerve cell injury involving large parts of the MCA territory [27, 50]. Immunohistological and immunofluorescence analyses for the presence of various leukocyte populations were investigated in coronal brain sections as described above using the antibodies listed in Table 2.

**Fig. 1:**
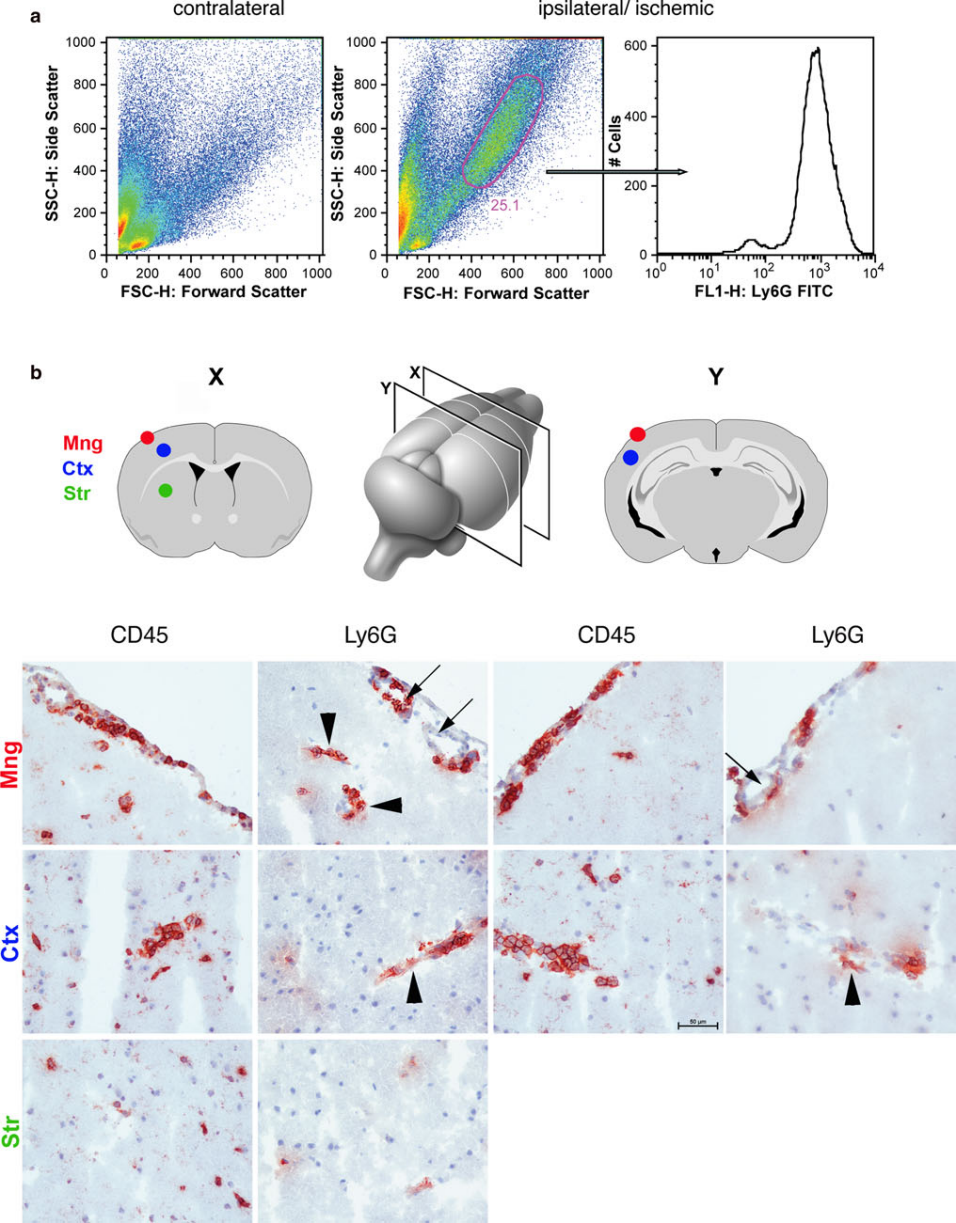
Localization of PMNs in the ischemic brain following 60 min of tMCAO and 18 h and 24 h reperfusion. **a** Inflammatory cells were isolated from the ipsilateral ischemic and contralateral hemisphere of 6 mice following 60 min tMCAO and 24 h reperfusion by enzymatic digestion and density gradient centrifugation, and analyzed by flow cytometry. Using Forward (FSC) and Side Scatter (SSC) profiles inflammatory cells were separated according to size (FSC) and granularity (SSC), respectively, and displayed in a *dot blot*. PMNs are characterized by a high SSC signal due to their high content of granules. Such a population was only found in the ipsilateral and not in the contralateral hemisphere. Positive Ly6G immunoreactivity of the scatter gated population, as depicted in the histogram (*right side*), confirmed their identity as PMNs. **b** Schematic representation of the two planes of the brain examined (*X* Bregma 0.50 mm, *Y* Bregma −2.46 mm) and corresponding coronal sections showing areas analyzed. Immunohistochemistry of sections from 60 min tMCAO and 24 h perfusion for CD45 and Ly6G reveal distribution of total leukocytes and PMNs, respectively, in the meninges (Mng), motor–sensory cortex (Ctx), and striatum (Str). The majority of cells were detected either intra- or perivascular in the meninges (*arrows*) and the penumbral cortex (*arrowheads*) and the cells comprised mainly Ly6G^+^ PMNs. *Bar* is 50 μm

There were no overt differences between C57BL/6 and Sv129 mice in the time point of first appearance or the distribution of CD45^+^ immune cells in the ischemic brains. Flow cytometry (Fig. 1a) and immunohistochemistry (Fig. 1b) revealed Ly6G^+^ PMNs to be the first cell type detectable in the ipsilateral hemisphere, independent of the duration of ischemia. However, the absolute time point of their appearance and how long they were detectable varied with occlusion length. The appearance of Ly6G^+^ PMNs was monophasic, with sparse appearance of PMNs at 12 h of reperfusion in 30 and 60 min tMCAO samples and at 3 h in 90 min tMCAO, in all cases peaking at 18–24 h (Fig. 1a).

Surprisingly, immunohistochemistry localized Ly6G^+^ cells predominantly to the leptomeningeal space of the ischemic hemisphere either confined within vessel lumina or closely associated with vessel surfaces regardless of MCA occlusion time (Fig. 1b). To a lesser extent, PMNs were also present in the leptomeninges of the contralateral hemisphere. Additional PMNs were detected in close association with arterioles in the motor–sensory cortex (Fig. 1b), and only isolated PMNs occurred in the striatum within the lesion core, principally in association with tears in the tissue (Fig. 1b). The latter was enhanced in 60 and 90 min occlusions where tissue integrity was severely compromised.

The only site where considerable numbers of PMNs were found outside of blood vessels was in the subarachnoid space covering the surface of both brain and optic nerves, remote from the ischemic area. PMNs were not observed in the adjacent cortical layers indicating their failure to penetrate the glia limitans. In accordance with previous reports [38], some PMNs were detected in the parenchyma of the lateral preoptic area (LPO) (Supplementary Fig. 1). As the LPO is not directly irrigated by the MCA these effects may be due to an indirect occlusion of the anterior medial striate artery that originates from the MCA [23] and irrigates dorso-lateral aspects of the caudate–putamen. The ophthalmic artery is derived from the internal carotid artery (ICA) that travels ventrally to the optic nerve within the optic canal and is enveloped within a dural sleeve of the optic nerve [40]. As the ICA is occluded in the tMCAO model employed, this probably accounts for the meningeal PMN accumulation observed around the optic nerve. Importantly, both the LPO and the meningeal infiltration around the optic nerve would skew flow cytometry or biochemical analyses for the presence of PMNs in the brain after experimental ischemic infarct.

Ly6C^+^CD11b^+^ monocytes were scarce and detectable at 24–48 h reperfusion in 30 and 60 min tMCAO; isolated CD45^+^CD11b^+^F4/80^+^ macrophages were detected at 48 h, peaking at 72 h reperfusion. In contrast to Ly6G^+^ PMNs, isolated Ly6C^+^CD11b^+^ monocytes were also found within the brain parenchyma at 24 h reperfusion, and at later stages also CD45^+^CD11b^+^F4/80^+^ macrophages (Supplementary Fig. 2a, b).

### Polymorphonuclear granulocyte (PMN) localization within the neurovascular unit (NVU)

**Fig. 2:**
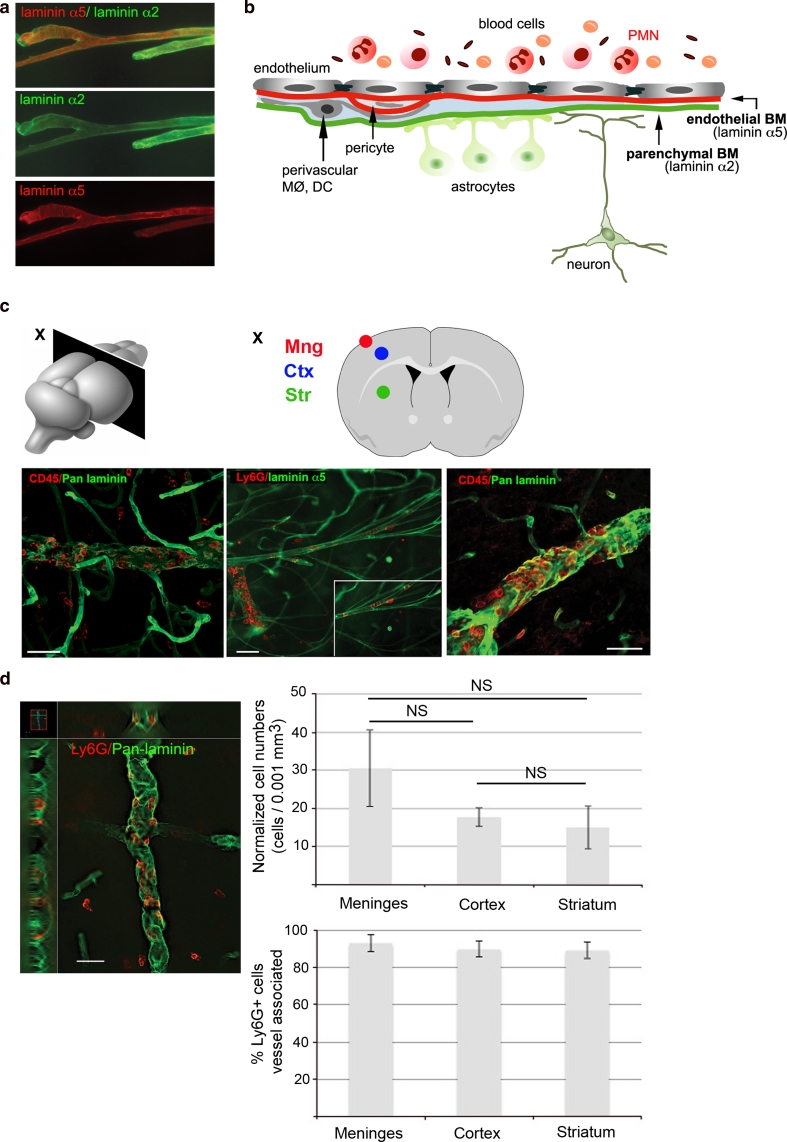
Localization of neutrophils in the neurovascular unit. **a** Confocal microscopy of a thick section (100 μm) double stained for laminin α5, showing the inner endothelial BM, and laminin α2, showing the outer parenchymal BM, and **b** schematic representation of the constituents of the NVU. Relative sizes and numbers of the NVU constituents are not to scale. MØ refers to macrophages and DC is dendritic cells. **c** Schematic representation of the plane of the brain examined (*X* Bregma 0.50 mm) and corresponding coronal section showing areas analyzed (striatum Str, meninges Mng and motor–sensory cortex Ctx). Corresponding confocal microscopy of thick sections from plane X of 60 min tMCAO, 24 h reperfusion, double stained with antibodies to pan-laminin, to mark all BMs, and CD45, or to laminin α5, as a marker of the endothelial BM, and Ly6G, reveal localization of total leukocytes and PMNs mainly in association with arteries or large arterioles (*outer panels*) and veins (*middle panel*). Images shown are from area Ctx. **d** Analyses of individual Z stacks permitted localization of Ly6G^+^ PMNs within vessel lumina or between the endothelial and parenchymal BM (vessel associated), or intra-parenchymally. Staining shown is for an arteriole. *Graphs* to the right show total Ly6G^+^ PMN numbers/0.001 mm^3^ brain volume normalized to the proportion of the brain volume occupied by vessels in *Str* striatum, *Mng* meninges and *Ctx* cortex. Normalized Ly6G^+^ PMN numbers associated with vessels in the different areas are expressed as percentages of the normalized total Ly6G^+^ cell numbers and show no statistically significant differences from 100 % cells associated with vessels (*P* values 0.56–0.86). Data shown are mean ± SEM from 2 to 3 mice. *NS* is no statistically significant difference. *Bars* in **c** and **d** are 40 μm

**Fig. 3:**
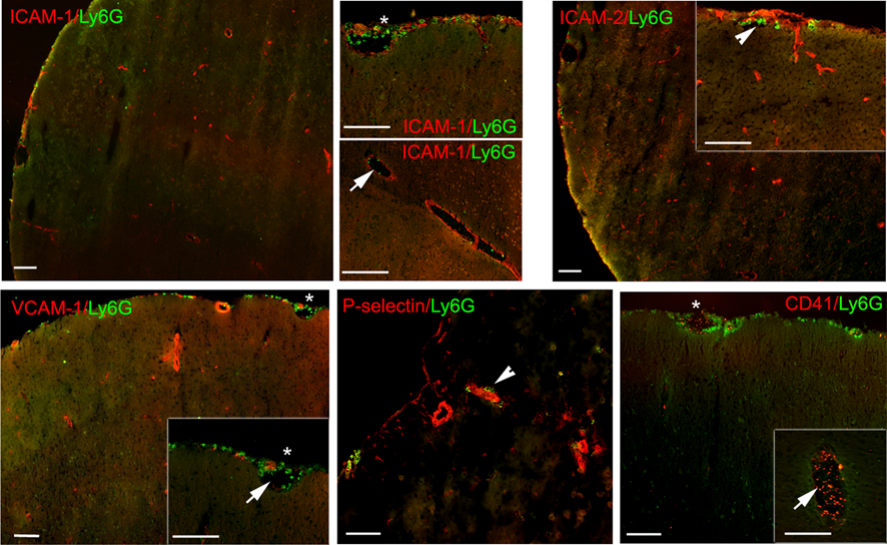
Endothelial adhesion molecules or platelet accumulation are not sufficient for Ly6G^+^ PMN extravasation in vivo. Data shown are for 60 min tMCAO and 24 h reperfusion (16 μm sections). Double immunofluorescence staining for Ly6G and intercellular adhesion molecule-1 (ICAM-1) and 2 (ICAM-2), vascular endothelial cell adhesion molecule (VCAM-1), or P-selectin showed some upregulation of adhesion molecules in cortical and meningeal microvessels of the ischemic hemisphere but no strict spatial correlation with Ly6G^+^ PMNs which localize intraluminally (*arrows*), perivascularly (*arrowhead*) or in the meningeal compartment (*asterisk*). Immunofluorescence staining for CD41^+^ platelets and Ly6G revealed that platelet accumulation in vessels was not associated with the localization of PMNs. *Bars* are 40 μm

Due to the limitations of immunohistochemistry and conventional immunofluorescence microscopy in localizing cells to defined layers within the NVU, fluorescently stained 100-μm sections were analyzed by confocal microscopy to obtain optical sections and 3D reconstructions. Thirty and 60 min tMCAO at 18 and 24 h reperfusion were examined. Sections were double stained with Ly6G or CD45 and pan-laminin antibody which recognizes the majority of the laminin isoforms, or a specific marker of the endothelial BM, laminin α5, permitting localization of PMNs within vessel lumina or in the perivascular space, as defined by the endothelial and parenchymal BMs (termed vessel associated), or outside of the endothelial and parenchymal BMs and hence within the CNS parenchyma (termed intraparenchymal) (Fig. 2a, b). Figure 2c illustrates that most CD45^+^ leukocytes and, specifically, Ly6G^+^ PMNs localized within the lumina of vessels of 30–40 μm diameter, predominantly representing arterioles, in the meninges and cortex, and did not occur in the ischemic core. Total numbers of CD45^+^ and Ly6G^+^ cells in the meninges, cortex, and striatum were counted and expressed per mm^3^ brain volume, and subsequently normalized to the proportion of the brain volume occupied by vessels, permitting assessment of the relative numbers of PMNs accumulated in the different brain areas, revealing an overall low number of PMNs and their tendency to localize to the meninges (although this was not statistically significant) (Fig. 2d). At 18 and 24 h reperfusion, numbers of CD45^+^ and Ly6G^+^ cells did not differ significantly and data shown in Fig. 2d are for Ly6G^+^ cells. Cells associated with vessels were similarly expressed per mm^3^ brain volume, and subsequently normalized to the proportion of the brain volume occupied by vessels. The percentage of vessel associated Ly6G^+^ cells was calculated using these normalized values (Fig. 2d). Statistical analyses revealed the absence of significant deviations from 100 % cells associated with vessels in the striatum (mean 90 % ± 7; *P* = 0.866), meninges (mean 94 % ± 4; *P* = 0.766), and cortex (mean 89 % ± 7; *P* = 0.56) at both 18 and 24 h reperfusion, regardless of occlusion time (data for 60 min tMCAO, 24 h reperfusion is shown in Fig. 2d). PMNs found were also not associated with CD41^+^ platelets (Fig. 3) or erythrocytes, indicating the absence of any hemorrhagic transformation.

### Polymorphonuclear granulocyte (PMN) accumulation is not associated with upregulated endothelial adhesion molecule expression, platelet aggregation or vascular permeability

PMN accumulation within blood vessel lumina suggests endothelial cell activation or aberrant endothelial cell physiology. To investigate potential correlations between PMN localization and local altered expression of endothelial cell adhesion molecules implicated in PMN adhesion to or migration across the vascular wall in inflammation, specifically P-selectin, ICAM-1, ICAM-2, and PECAM-1 were analyzed in 60 min tMCAO samples at 6, 12, 18, 24 h of reperfusion using confocal immunofluorescence microscopy. No differences in staining patterns or intensity were observed for PECAM-1 between lesioned and non-lesioned hemispheres (not shown). As previously reported, immunofluorescence staining for vascular ICAM-1 and VCAM-1 was more extensive in the ischemic area [16, 51, 63], principally in vessels in the meninges and cortex (Fig. 3). Staining for P-selectin, which is not constitutively expressed in the brain parenchyma [22], was enhanced in isolated vessels in the penumbra at 12 h and 24 h reperfusion (Fig. 3). Despite enhanced immunofluorescence staining for P-selectin, ICAM-1 and VCAM-1 in many vessels of the lesioned hemisphere compared to the non-lesioned hemisphere, there was no strict spatial correlation of increased adhesion molecule expression with sites of vascular PMN accumulation and >90 % of the vessels showing increased VCAM-1 and ICAM-1 staining were not associated with Ly6G^+^ cells (Fig. 3) nor was PMN localization associated with platelet aggregates, as determined by double immunofluorescence staining for Ly6G and CD41 (Fig. 3). Rather, platelet aggregations were localized mainly to the lumina of dilated vessels both in the absence and presence of PMNs (Fig. 3). Finally, neutrophil extracellular traps (NETs), which have been reported to trap PMNs within vessels lumens, were investigated by staining for DNA as previously described [85], revealing the absence of vascular NET formation in any of the samples analyzed.

Importantly, PMN accumulation within blood vessels was not associated with local changes in vascular permeability as shown by triple staining for murine immunoglobulin (IgG), Ly6G and pan-laminin (Supplementary Fig. 3a). In general, the presence of IgG in the brain parenchyma was detected in 40 % of all 60 min tMCAO 18 h and 24 h samples, mostly observed around larger vessels in the cortex, and was not correlated with sites of Ly6G^+^ PMN accumulation (Supplementary Fig. 3a). To further address possible in vivo poststroke BBB leakage, 60 tMCAO/24 h reperfusion mice were intravenously injected with either Evans Blue and Hoechst 33258 or 3 kDa Texas Red-Dextran and 10 kDa FITC-Dextran prior to sacrifice. In addition to staining of endothelial cell nuclei, Hoechst 33258 labelled nuclei of neurons in the lateral preoptic area (LPO) of the ischemic hemisphere. Extravasation of Evans blue (Supplementary Fig. 3b) or labelled dextrans (data not shown) was furthermore observed only around some dilated vessels within the striatum or around focal arterioles in the penumbra in the ischemic hemisphere.

### Oxygen and glucose deprivation fails to induce polymorphonuclear granulocyte (PMN) extravasation across the blood–brain barrier under physiological flow in vitro

**Fig. 4:**
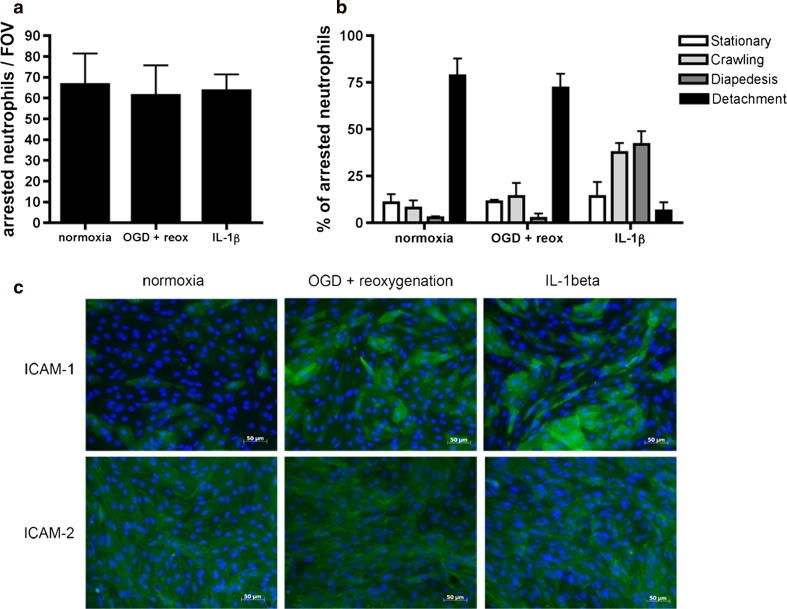
PMN interaction with endothelium in an in vitro blood–brain barrier model. PMN interaction with pMBMECs under normoxic, ischemic (OGD + reox) or IL-1β stimulated conditions under physiological flow (1.5 dyn/cm^2^) was recorded for 20 min and the dynamic behaviour of arrested PMNs was analyzed. **a** The number of PMNs arrested on pMBMECs counted per field of view (FOV). **b** Proportions of PMNs that were stationary, crawling, undergoing diapedesis or detachment from pMBMECs. PMNs that remained immobile on the monolayer were defined as ‘Stationary’, PMNs that polarized and crawled on the monolayer but did not diapedese across the endothelial monolayer were described as ‘Crawling’, PMNs that crawled until they found a suitable site for diapedesis were defined as undergoing ‘Diapedsis’, and PMNs that detached during the video acquisition time were termed ‘Detachment’. Data in **a** and **b** are mean ± SD, *n* = 3. **c** Immunofluorescence staining of pMBMECs for ICAM-1 and ICAM-2 under normoxic, ischemic, and IL-1β stimulated conditions shows upregulation of ICAM-1 under both, ischemic and IL-1β stimulation, ICAM-2 staining remains unaffected. Endothelial cells are counter-stained with Hoechst dye to show the cell nuclei. *Bar* 50 μm

Our observations suggest that in contrast to inflammatory stimuli, ischemia/reperfusion fails to induce the signals in brain endothelial cells required to mediate the extravasation of circulating PMNs into the brain parenchyma. To mimic PMN interactions with the BBB under physiological flow (1.5 dyn/cm^2^) after ischemia/reperfusion in vitro, glial cells in coculture with primary mouse brain microvascular endothelial cells (pMBMECs) were exposed to normoxic conditions or 4 h of oxygen and glucose deprivation (OGD) followed by 20 h of reoxygenation. For live cell imaging, the endothelial monolayer was placed in the flow chamber and highly purified bone marrow derived PMNs were perfused over the pMBMECs. The dynamic PMN interaction with the pMBMECs was recorded under constant flow for 20 min. Comparisons were made with cocultures treated with or without IL-1β under normoxic conditions. While PMNs were able to arrest, crawl, and diapedese across the IL-1β treated pMBMECs, pMBMECs exposed to OGD/reoxygenation induced brief PMN arrest and crawling but did not result in PMN diapedesis across the endothelium, thus resembling non-stimulated normoxic conditions (Fig. 4a, b; Supplementary videos 1–3). Surprisingly, OGD/reoxygenation upregulated cell surface expression of adhesion molecules, as exemplified by the increased immunostaining for ICAM-1 on the pMBMECs (Fig. 4c), which was similar to that induced by IL-1β treatment. These data indicate that although ICAM-1 is upregulated on brain endothelium under ischemic conditions in vitro and in vivo, ischemia/reperfusion fails to induce the sum of traffic signals in brain endothelial cells required to promote PMN transmigration across the endothelial monolayer.

### Localization of polymorphonuclear granulocytes (PMNs) in human stroke specimens

**Fig. 5:**
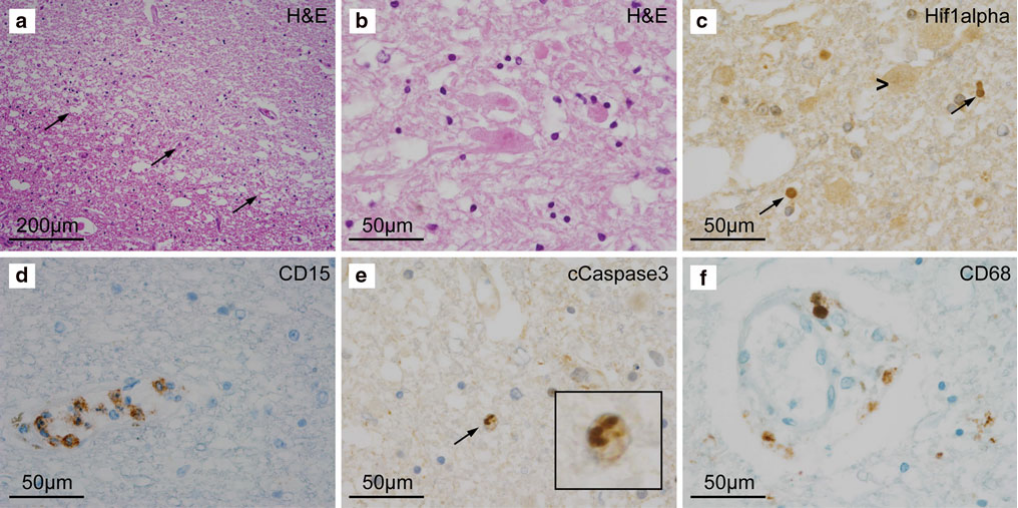
Histopathology of human acute stroke specimens (Stage I). **a** H&E staining of human stage I stroke specimen demonstrating demarcation of the ischemic core from the penumbra (*arrows*); **b** H&E staining (high magnification of **a**) showing the presence of eosinophilic neurons indicating an early ischemic neuronal damage; **c** At the infarct border zone, eosinophilic neurons (*arrowhead*, as depicted in Fig. 5b) are surrounded by glial cells showing severe hypoxic changes (*arrows*) as indicated by the strong expression of hypoxia-inducible factor-1 alpha (Hif1alpha); **d** CD15 immunohistochemistry indicating that PMNs are primarily located within blood vessels in acute human stroke lesions; **e** characterization of cells with histomorphological features of PMNs within the CNS parenchyma: while intravascular cells with PMN-like morphology (as seen in Fig. 5d) were strongly CD15-positive, intraparenchymally located cells exhibiting PMN-like morphology and being CD15-negative are strongly positive for cleaved caspase-3 (*arrow*) indicating that these cells undergo apoptosis; **f** CD68-positive cells of the monocytic lineage are mainly located in the perivascular space or within the brain parenchyma. Data shown are from a 75-year-old male patient suffering from an acute right parietal ischemic infarct (for details see Supplementary Table 1)

To determine whether our findings were relevant to human stroke, 25 specimens [24 autopsy cases including 17 pure acute stroke (stage I, Fig. 5) and 8 mixed (apart from stage I also showing stage II or III infarct regions in other CNS areas) and one biopsy specimen (Supplementary Table 1)] were examined by immunohistochemistry and immunofluorescence microscopy as described for the mouse tMCAO samples. As anti-human Ly6G antibodies are not available, morphology together with CD15^+^ immunostaining (which mainly recognizes PMNs and a subset of monocytes) and enzyme histochemistry for myeloperoxidase and chloracetate esterase were employed to identify PMNs. Very few PMNs were detected in both early infarct stages (stage I) and at stages of resorption (stage II) (Supplementary Fig. 4), with the majority of the PMNs being localized either within the lumen of blood vessels (Fig. 5d) or in the perivascular space between the endothelial and parenchymal BMs visualized using a pan-collagen type IV antibody (data not shown). Absence of granulocytic infiltration into the CNS parenchyma was especially noted in very acute stroke lesions (<48 h), even though this has been proposed to be the main time frame for PMNs to invade infarcted brain tissue after CNS ischemia. Analyses of samples of such <48 h infarct lesions revealed the appearance of cells morphologically resembling PMNs not only in vessels but also in the CNS parenchyma; however, CD15 staining was restricted to cells within vessel lumina (Fig. 5d). Indeed, upon careful examination multiple cells showing PMN morphology in the CNS parenchyma were found to be positive for cleaved caspase-3 (Fig. 5e), suggesting that they represent apoptotic bodies, which morphologically are easily confused with PMNs due to their fragmented nuclei. Already at this early infarct stage, a low amount of extravasated CD68^+^ monocytic cells was observed (Fig. 5f). Using this combination of CD15 staining, together with morphology and enzyme histochemistry for myeloperoxidase and chloracetate esterase (not shown) to identify PMNs in very early infarct lesions, rare infiltration in the subarachnoid and the subpial space, in the cortical layers I and II (not shown) and the Virchow-Robin space was observed. No PMNs were detected in the inner cortical layers or in the infarct center and border zones. Even at later stages after infarction PMNs remained confined to vessel lumina, despite extensive presence of CD45-positive leukocytes (not shown) which mainly consisted of CD163-negative (not shown) and CD68-positive macrophages and activated microglia and a moderate fraction of CD3-positive T-cells (while CD20-positive B-cells were virtually absent) and aberrant dilated appearance of vessels (Supplementary Fig. 4), indicative of ischemia.

## Discussion

By bringing together stroke researchers, neuropathologists, cell biologists, and neuroimmunologists specialized on the cellular and extracellular matrix components of the NVU and immune cell penetration of the NVU, we have been able to comprehensively investigate the in vivo PMN localization after ischemic stroke in mouse and human samples. Our data support an early appearance of PMNs after ischemic stroke in both mouse tMCAO and in human samples, as shown by others [21], but contrary to previous concepts our data show that PMNs are (1) limited in number, (2) associate with vessel lumina or the perivascular and leptomeningeal space, and (3) do not strictly correlate with either platelet aggregates, sites of increased vessel permeability, or sites of enhanced expression of endothelial adhesion molecules known to be required for PMN extravasation in inflammation. Studies of CNS autoimmune inflammation have shown that localization of immune cells within the leptomeningeal and perivascular space is not sufficient to induce disease symptoms [1, 8]. Rather, penetration of the parenchymal BM is required before access to CNS parenchyma and induction of pathological processes is possible [1, 84]. Hence, the localization of PMNs to the vasculature early after ischemic stroke necessitates reassessment of their role in stroke.

Although some adhesion molecules, such as VCAM-1, were upregulated on vessels in the ischemic hemisphere, the expression of adhesion molecules was heterogeneous with some vessels having low and others high expression levels, and there was no spatial correlation with PMN accumulation within vessels or in the perivascular space. While previous studies have investigated adhesion molecules in ischemic stroke showing results similar to those obtained here [16, 51, 63], no previous study has correlated in vivo adhesion molecule expression with localization of PMNs. Indeed, most studies have involved flow cytometry or myeloperoxidase expression in excised brains to quantify PMNs while adhesion molecules were analyzed by immunofluorescence microscopy on tissue sections, which led to the false conclusion that the two are correlated. This has also been the justification for employing mice lacking ICAM-1, or the use of function blocking antibodies targeting adhesion molecules in MCAO experiments, which have produced variable results [9, 36, 47]. Normally, extravasation of PMNs during inflammation occurs at the level of postcapillary venules [69] and involves E- and P-selectin-mediated rolling on the endothelial cell surface, and subsequent ICAM-1 mediated arrest and diapedesis across the endothelial cell monolayer [31, 55]. The absence of a spatial correlation between upregulated expression of endothelial P-selectin, VCAM-1 and ICAM-1 and vascular sites of PMN accumulation in the tMCAO samples suggests the absence of the complete cascade of these events and that the mode of endothelial activation that occurs after ischemic stroke is not sufficient to trigger PMN extravasation into the brain parenchyma. This is supported by the in vitro studies involving pMBMECs, which demonstrated that while OGD/reoxygenation can upregulate endothelial ICAM-1 this was not sufficient to support transmigration of PMNs across the pMBMEC monolayer. In addition, the failure of those few PMNs that enter the perivascular space to penetrate into the brain parenchyma proper also reflects the absence of the molecular signals required for their invasion into the CNS as observed in inflammation.

The concept that the brain parenchyma is a tissue that is unique in its resistance to leukocyte diapedesis has previously been suggested by others, who have shown that even direct intracerebral injection of chemotactic cytokines that are sufficient to induce PMN extravasation into other tissues fail to trigger PMN extravasation into the brain parenchyma [4]. Thus, PMN migration from the blood stream across the BBB and the glia limitans into the brain parenchyma requires more than presence of chemotactic factors and induction of leukocyte adhesion to cerebral endothelium.

The precise molecular mechanism involved in PMN accumulation within vessels observed in the current study is not clear and we can only speculate on the molecules involved. The fact that PMNs accumulated in larger vessels, mainly arterioles, further supports the hypothesis that the molecular mechanism/s involved are distinct from those described above for postcapillary venules. A recent study involving transient ligation and reperfusion of the vena cava reported a similar PMN accumulation that was not associated with expression of the classical adhesion molecules or platelets, but rather with DNA NETs released by PMNs and resulting in their aggregation [85]. This was not the case here, as NETS were not detected in any of the tMCAO samples. However, it is possible that hypoxia or changes in blood flow, resulting from vessel occlusion, alters the expression of as yet unidentified adhesion molecules, either upregulating molecules that promote PMN adherence but also downregulating anti-adhesive molecules. The endothelial glycocalyx, composed largely of glycosaminoglycans carrying highly negatively charged heparin sulfate chains, is known to repel cells in the circulation, and the depletion of heparan sulfate chains significantly enhances immune cell adhesion [57, 58]. As the glycocalyx changes with blood flow [42], it is conceivable that it may present part of a novel adhesion mechanism in larger vessels. This is supported by the recent identification of a role for myeloperoxidase, released from PMNs, in charged interactions between PMNs and the gylcocalyx of endothelium which promotes PMN adherence [53]. While this is a promising field, the role of charge molecules such as cell surface glycans has rarely been considered in immune cell recruitment, mainly due to the complex chemistry associated with their analysis.

Importantly, our data provide evidence that previous studies have overestimated PMN contribution to stroke because of the methods used for their identification and shed new light on previous animal studies and clinical trials. The largest majority of previous studies have focused on permanent MCAO, rather than the temporary MCAO employed here, which may be one reason for the comparatively low PMN numbers detected in our study. In addition, the high numbers of PMNs detected in the leptomeningeal areas, especially surrounding the optical nerve as detected here but also reported previously [38], suggest that previous studies solely based on flow cytometry or enzymatic assessment of PMN infiltration into the brain are likely to have equated these findings with number of PMNs present within the ischemic brain parenchyma. Myeloperoxidase, commonly used as a PMN marker, is expressed by PMNs, monocytes and activated microglia present in the ischemic brain [13], while the Gr-1 antibody (clone RB6-8C5), frequently used to identify PMNs, recognizes both Ly6C and Ly6G expressed by monocytes and PMNs, respectively [35]. The use of these tools would therefore inevitably lead to an over-estimation of PMNs. Only few studies have attempted to localize PMNs in the brain parenchyma at defined times after MCAO, including that of Garcia and Kamijyo [38], who employed electron microscopy of permanent MCAO in rats. Consistent with our data, the majority of the PMNs were found in vessels, and while rare isolated PMNs were detected outside of the endothelial layer, the magnifications shown are too high to definitively state that the PMN is in the brain parenchyma (and not in the perivascular space) nor do they provide evidence for neuronal death in the close vicinity of PMNs, rather isolated images of damaged neurons are shown. The concept that PMNs contribute to reperfusion injury at early stages after ischemic stroke has led to clinical trials targeting PMNs to minimize infarct volume, most of which have not been successful [25]. While this may in part be attributable to factors such as patient cohort size or demography, or adverse effects of the PMN targeting strategies [36], the data presented here suggest that the role of PMNs in ischemic stroke remains unclear and needs further investigation before their consideration as a relevant therapeutic target.

The PMN specific anti-Ly6G antibody (clone 1A8) employed here [17], in conjunction with immunofluorescence staining for defined markers of the vascular basement membranes and confocal analyses have permitted a reliable identification and localization of PMNs in ischemic brain sections, and highlight the importance of supplementing quantitative analyses of ischemic tissue by in vivo localization studies. However, the choice of such in vivo imaging technologies is also crucial; while intravital microscopy (IVM) via a cortical window has been used to assess in vivo leukocyte–endothelial cell interactions in the ischemic brain [5, 48], the working depth achievable is limited and in addition only meningeal postcapillary venules run parallel to the surface of the skull, whereas cortical postcapillary venules are orientated perpendicularly to the surface of the brain and the focal plane [46, 71]. Hence, it is impossible to discern leukocyte–endothelial interactions in cortical postcapillary venules as this requires tracing of leukocytes over a defined period along a certain vessel length. As meningeal and parenchymal blood vessels differ from one another in several aspects [64], including the absence of a glial ensheathment in leptomeningeal vessels [70] and the constitutive expression of P-selectin in meningeal but not parenchymal microvessels [6], studying meningeal vessels in stroke is not a substitute for cortical microvessels. Given our detection of a high number of PMNs in the leptomeninges, intravital imaging of such areas might overestimate PMN recruitment into the brain after stroke. Taken together, this suggests that confocal microscopic analysis of thick brain sections using defined markers of immune cells and the borders of the NVU is, at present, the most efficient mode of assessing immune cell infiltration after ischemic stroke in experimental models.

The analysis of human autopsy samples corroborated the observation that PMNs rarely occur in the brain parenchyma early after stroke. Clearly, it is impossible to prove that the patient samples analyzed had undergone reperfusion after the vessel occlusion/brain ischemia and to define this histologically. However, spontaneous reperfusion is a frequent phenomenon in clinical stroke, which may also occur in parts of the ischemic area, and even in patients receiving thrombolysis often some brain tissue undergoes infarction despite reperfusion [43, 74]. Hence, a spectrum ranging from no reperfusion to different times and extent of reperfusion are likely in such autopsy samples, validating the comparison with the mouse tMCAO data. The question therefore arises how the concept that PMN leukocytes strongly infiltrate the brain parenchyma within the first 24 h after onset of brain infarction has gained access into widely accepted neurological and neuropathological textbooks [33]. Several studies have defined the time frame of PMN detection in the brain after ischemic stroke, mainly using SPECT and CT scan analyses of injected tracer immune cells [2, 87]. Already these studies revealed conflicting results about when and over which time period PMNs are detectable in the brain; however, the discrepancies were attributed to the injection of mixed leukocytes [87] versus purified PMNs prior to SPECT [2]. While SPECT permits high temporal resolution of such tracer cells, it has poor spatial resolution and does not permit the distinction between PMNs within or outside the confines of the NVU. A second point of conflict might be the extent of foci of fresh bleedings in mainly ischemic infarction. Even selected pictures from classical textbooks [33], employed as evidence for PMN entry into the brain parenchyma after ischemic stroke, depict fresh foci of hemorrhages and associated leakage of various cellular populations including erythrocytes and monocytes, indicative of more severe vascular disruption. Therefore, vascular disruption might be a very logical explanation for the detection of PMNs in the CNS parenchyma in stroke cases accompanied by hemorrhages. However, these findings differ considerably from classical forms of purely ischemic stroke lesions. Finally, pioneering studies addressing the role of PMNs in stroke did not have access to the complex panel of immunocytochemical markers, available now, to precisely define cell types and their localization in relation to the cellular and BMs layers of the NVU, but rather relied mainly on classical histomorphological examination [38, 79]. These drawbacks might constitute problems if sections are assessed by histology only where PMNs may be confused with different stages of apoptotic figures, which also frequently occur in hypoxic-ischemic brain lesions, due to their similar morphology. While isolated studies exist, like that of Lindsberg et al. [56], which included early human stroke samples without secondary hemorrhages showing CD15-positive cells in the lesioned CNS, even these state that granulocytes in early infarction are mostly found within the intravascular space and typically aggregate at the walls of CNS vessels and are, therefore, in accordance with our findings. However, Lindsberg et al. admit that a systematic evaluation of morphological details such as whether granulocytes were still surrounded by a capillary lumen was not possible in their cohort.

In conclusion, our data indicate that PMNs do not gain access to the brain parenchyma early after ischemic stroke and highlight the vascular compartment of the NVU rather than neurons, as previously suggested also by others [20], as the site of potential PMN action.

## Electronic supplementary material

Below is the link to the electronic supplementary material.

Supplementary material 1 (DOCX 84.5 kb)

**Supplementary Figure 1** **Moderate immune cell accumulation following 60 min tMCAO and 18h reperfusion.** H&E staining of coronal CNS sections illustrating areas analysed by immunohistochemistry. Most CD45^+^ cells and Ly6G^+^ PMNs localized to the meninges (Mng), blood vessels in the cortex (Ctx) and to the lateral preoptic area (Lpo). Detection of PMNs in the lateral preoptic area (Lpo) always coincided with destruction of blood vessels and the loss of tissue integrity. Bars are 20 μm. (TIFF 25513 kb)

**Supplementary Fig 2. Immunofluorescence staining for PMNs, monocytes and macrophages in 60 min tMCAO samples**. A) Immunofluorescence staining for pan-laminin to mark the border of the NVU together with Ly6G reveals that at 24h after ischemia/reperfusion PMNs localize predominantly to vessel lumina in the meninges and cortex, while at the same time point Ly6C+ monocytes although scarce occasionally appear in the brain parenchyma (arrow). b) At 72h, F4/80+ macrophages /activated microglia are abundant within and outside vessels. Bars are 70 μm. (TIFF 2977 kb)

**Supplementary Fig 3. Ly6G** ^**+**^ **PMN accumulation does not correlate with increased vascular permeability.** 60 min tMCAO at 24 h reperfusion data are shown. a) Two upper panels show contralateral and ipsilateral brain hemispheres triple stained for mouse IgG, to visualize serum protein extravasation into the CNS parenchyma, Ly6G^+^ PMNs and pan-laminin to mark the border of the NVU; nuclei are visualized by DAPI. Lower panels show single stainings of the vessels marked by the arrows in the panels immediately above. The arrows mark positions of IgG staining alone or in association with PMNs within vessel lumina, showing no strict correlation between Ly6G^+^ cells and IgG. Bar is 40 μm. b) Perfusion with Hoechst 33258 and Evans Blue revealed the integrity of the majority of the BBB microvessels in the ischemic striatum and penumbra (top row, bars are 100 μm). On rare occasions, Evans Blue penetrated dilated vessels in the ischemic hemisphere where diffuse perivascular extravasation is seen beyond the Hoechst-labeled nuclei of the endothelial cell layer; Lpo is in the lateral preoptic area (bottom row shows high magnification, bars are 50 μm). The permeability marker remained strictly confined to the vessel lumen in the contralateral hemisphere. Similar results were observed in the Dextran infused mice (not shown). (TIFF 8940 kb)

**Supplementary Fig 4. Histopathology of human subacute stroke specimens (Stage II).** (left upper) H&E stain indicating macrophage-rich lesion (see insert for higher magnification) which is sharply delimited from the surrounding penumbra (scale bar: 1mm); (right upper) the largest cell population in subacute human stroke lesions consists of CD68-positive macrophages/microglia (scale bar: 100µm); (left, lower) CD15-positive PMNs were virtually absent in those lesions while still being present within blood vessels (asterisk; scale bar: 100µm); (right lower) a moderate number of CD3-positive T-lymphocytes is also present in this subacute human stroke lesion (scale bar: 100µm). Data shown are from a 45-year-old male patient suffering from a large ischemic infarct within the territory of the right middle cerebral artery (for details see Supplementary Table 1). (TIFF 4697 kb)

**Supplementary Video 1. PMN interaction with normoxic blood-brain barrier endothelium under flow.** One representative movie corresponding to experiments evaluated in Fig. 4 a,b showing that perfused PMNs arrest on the apical surface of the normoxic BBB monolayer and start crawling in the direction of the flow, with most cells detaching during the 20 min acquisition time. Brain microvascular endothelial cells were seeded on Millicell CM filters impairing the clear visualization of the endothelial monolayer. Arrested PMNs appears as phase-contrast bright cells. Time-lapse movie was acquired under phase-contrast illumination at x20 magnification using a monochrome CCD camera (AxioCam MRm Rev, Zeiss). Images were taken every 20 sec and the movie is composed at 7 frames per second (fps) (140 fold speed enhancement). Scale bar is 50 µm. (MPEG 2560 kb)

**Supplementary Video 2. PMN interaction with the ischemic blood-brain barrier endothelium under flow.** One representative movie corresponding to experiments analysed in Fig. 4 a,b is shown. PMN interaction with the ischemic endothelium shows the same pattern as with normoxic endothelium, where arrested PMNs start crawling on top of the endothelial monolayer but detach after several minutes. For detailed description of the video acquisition see supplementary video legend 1. (MPEG 2532 kb)

**Supplementary Video 3. PMN interaction with 4h IL-1β-stimulated blood-brain barrier endothelium under flow.** One representative movie corresponding to experiments evaluated in Fig. 4 a,b. On brain microvascular endothelial cells pre-treated with IL-1β, arrested neutrophils crawl (phase-contrast bright) and diapedese across the BBB monolayer (phase-contrast dark) where they continue to move beneath the endothelium. Yellow arrows follow two phase-bright neutrophils crawling on the BBB endothelium under flow. White arrows indicate the precise site of diapedesis. Blue arrows track the phase-dark neutrophils moving underneath the monolayer. For detailed description of the video acquisition see supplementary video legend 1. (MPEG 2578 kb)
