# Supplementary material for: The neurovascular unit as a selective barrier to polymorphonuclear granulocyte (PMN) infiltration into the brain after ischemic injury
Source: Acta Neuropathol. 2012 Dec 27;125(3):395–412. doi: 10.1007/s00401-012-1076-3 (PMC3578720; doi:10.1007/s00401-012-1076-3)
Supplement: Supplementary file 1 — Supplementary material 1 (DOCX 84.5 kb) [file 401_2012_1076_MOESM1_ESM.docx]

**Supplementary Table 1: Human stroke specimens analysed**

| **Age** | **Sex** | **Survival after stroke onset** | **Main clinical diagnoses** | **Cause of death** | **Neuropathological diagnoses** | **Autopsy/**  **Biopsy** |
| --- | --- | --- | --- | --- | --- | --- |
| 67 | F | 6 h | - coronary heart disease  - aortic dissection | cerebral infarction | - ischemic infarct (stage I) | A |
| 65 | F | 10 h | - chronic venous insufficiency  - epilepsy | cerebral infarction | - ischemic infarct (stage I) | A |
| 62 | F | 12 h | - arterial hypertension  - depression  - pneumonia  - colitis | myocardial infarction | - ischemic infarct (stage I) | A |
| 72 | M | 11 h | - diabetes mellitus  - nephropathy  - cardiac arrhythmia  - cardiac insufficiency | myocardial infarction | - ischemic infarct (stage I) | A |
| 60 | M | 12 h | - aortic dissection | aortic bleeding | - right parietal infarct (stage I) | A |

| **Age** | **Sex** | **Survival after stroke onset** | **Main clinical diagnoses** | **Cause of death** | **Neuropathological diagnoses** | **Autopsy/**  **Biopsy** |
| --- | --- | --- | --- | --- | --- | --- |
| 45 | F | 24 h | - lymphangioleiomyomatosis | pulmonary bleeding | - large infarct of right hemisphere (stage I)  - severe brain edema | A |
| 86 | F | 24 h | - cardiac arrhythmia  - colitis | acute heart failure | - ischemic infarct (stage I) | A |
| 62 | M | 48 h | none | circulatory arrest | - ischemic infarct (stage I) | A |
| 56 | F | 60 h | - atrial fibrillation  - cardiac insufficiency  - asthma  - obesity  - alcohol and nicotin abuse | circulatory arrest | - ischemic infarct (stage I) | A |
| 78 | F | 60 h | - arteriosclerosis  - pneumonia | cerebral infarction | - ischemic infarct (stage I) | A |
| 68 | M | 72 h | - muscular atrophy | cerebral infarction | - ischemic infarct (stage I) | A |

| **Age** | **Sex** | **Survival after stroke onset** | **Main clinical diagnoses** | **Cause of death** | **Neuropathological diagnoses** | **Autopsy/**  **Biopsy** |
| --- | --- | --- | --- | --- | --- | --- |
| 74 | F | 72 h | - tachyarrhythmia  - arterial hypertension  - arteriosclerosis | cerebral infarction | - ischemic infarct (stage I) | A |
| 73 | F | 72 h | - diabetes mellitus  - arterial hypertension | myocardial infarction | - ischemic infarct (stage I) | A |
| 47 | M | 7 d | - myocardial infarction  - pneumonia | heart failure (after reanimation) | - severe brain edema  - hypoxic encephalopathy  - ischemic tectal infarcts (stage I-II) | A |
| 75 | M | 7 d | - acute myeloid leukemia  - ischemic infarct/ right hemisphere  - Hypertension  - prostate cancer since 11/05  - dementia symptoms | thrombocytopenia due to AML, multimorbidity | - multiple ischemic microinfarcts (stage I) left frontal, parietal, occipital and temporal cortex  - ischemic infarct (stage II) right basal ganglia | A |

| **Age** | **Sex** | **Survival after stroke onset** | **Main clinical diagnoses** | **Cause of death** | **Neuropathological diagnoses** | **Autopsy/**  **Biopsy** |
| --- | --- | --- | --- | --- | --- | --- |
| 56 | M | NA | - pharmaco resistent right temporal lobe epilepsy since childhood | Does not apply  (epilepsy surgery) | - hippocampal sclerosis  - focal cortical dysplasia (Palmini 2a)  - ischemic infarct (stage I) right temporal cortex | B |
| 57 | M | 3 w | - coronary heart disease  - heart failure  - hypoxic brain damage after reanimation | respiratory insufficiency after pneumonia | - laminar cortical necrosis/hypoxic encephalopathy  - right parieto-occipital ischemic infarct (stage I-II)  - severe brain edema | A |
| 50 | F | 3 w | - congenital heart defect (truncus arteriosus communis typ IV) | cardiac insufficiency due to the underlying heart disease | - subarachnoid hemorrhage  - ischemic infarct (stage I) cortex left fronto-parietal  - ischemic infarct (stage II) right frontal cortex | A |

| **Age** | **Sex** | **Survival after stroke onset** | **Main clinical diagnoses** | **Cause of death** | **Neuropathological diagnoses** | **Autopsy/**  **Biopsy** |
| --- | --- | --- | --- | --- | --- | --- |
| 45** | M | 2 m | - infarction of the middle cerebral artery  - myocardial infarction | malignant brain edema | - large ischemic infarct within the territory of the right middle cerebral artery and secondary fresh bleedings  - severe brain edema | A |
| 61 | M | Appr. 4 m | - septic cardiomyopathy  - coronary heart disease  - coronary bypass  - stenosis of the internal carotid artery | heart failure  (after reanimation) | - hypoxic encephalopathy  - ischemic infarct in the medulla oblongata (stage I)  - ischemic infarcts (stage II-III) in the mesencephalon and the right cerebellum | A |
| 61 | F | ND | - sarcoidosis  - endocarditis  - renal failure  - liver failure | septic pneumonia | - left parietal ischemic infarcts (stage I-II) with bleedings and astrogliosis  - right parietal ischemic infarcts (stage I-II) | A |

| **Age** | **Sex** | **Survival after stroke onset** | **Main clinical diagnoses** | **Cause of death** | **Neuropathological diagnoses** | **Autopsy/**  **Biopsy** |
| --- | --- | --- | --- | --- | --- | --- |
| 71 | F | ND | - renal failure  - multiple thromboses | cerebellar infarcts with increased intracranial pressure | - ischemic infarcts (stage I) in both cerebellar hemispheres and secondary fresh bleedings  - brain edema | A |
| 81 | M | ND | - aortic valve stenosis  - coronary heart disease  - coronary artery bypass | electromechanical dissociation after surgery | - ischemic infarcts (stage I-II) within the territory of the middle cerebral artery  - macroangiopathy & microangiopathy | A |
| 77 | F | ND | - glioblastoma WHO grade IV, left central region  - arterial hypertension | epileptic state | - glioblastoma WHO grade IV, left central region  - right occipital and right frontal ischemic infarcts (stage I) | A |
| 75* | M | ND | - combined insufficiency of mitral, tricuspid and aortic valves | multi-organ failure | - right parietal ischemic infarct (stage I)  - moderate hypoxic encephalopathy  - meningioma, WHO grade I | A |

NA is not applicable; ND is not determined. *Patient depicted in Fig. 5; **Patient depicted in Supplementary Fig. 4.
