# Supplementary figures and images for: The neurovascular unit as a selective barrier to polymorphonuclear granulocyte (PMN) infiltration into the brain after ischemic injury
Source: Acta Neuropathol. 2012 Dec 27;125(3):395–412. doi: 10.1007/s00401-012-1076-3 (PMC3578720; doi:10.1007/s00401-012-1076-3)

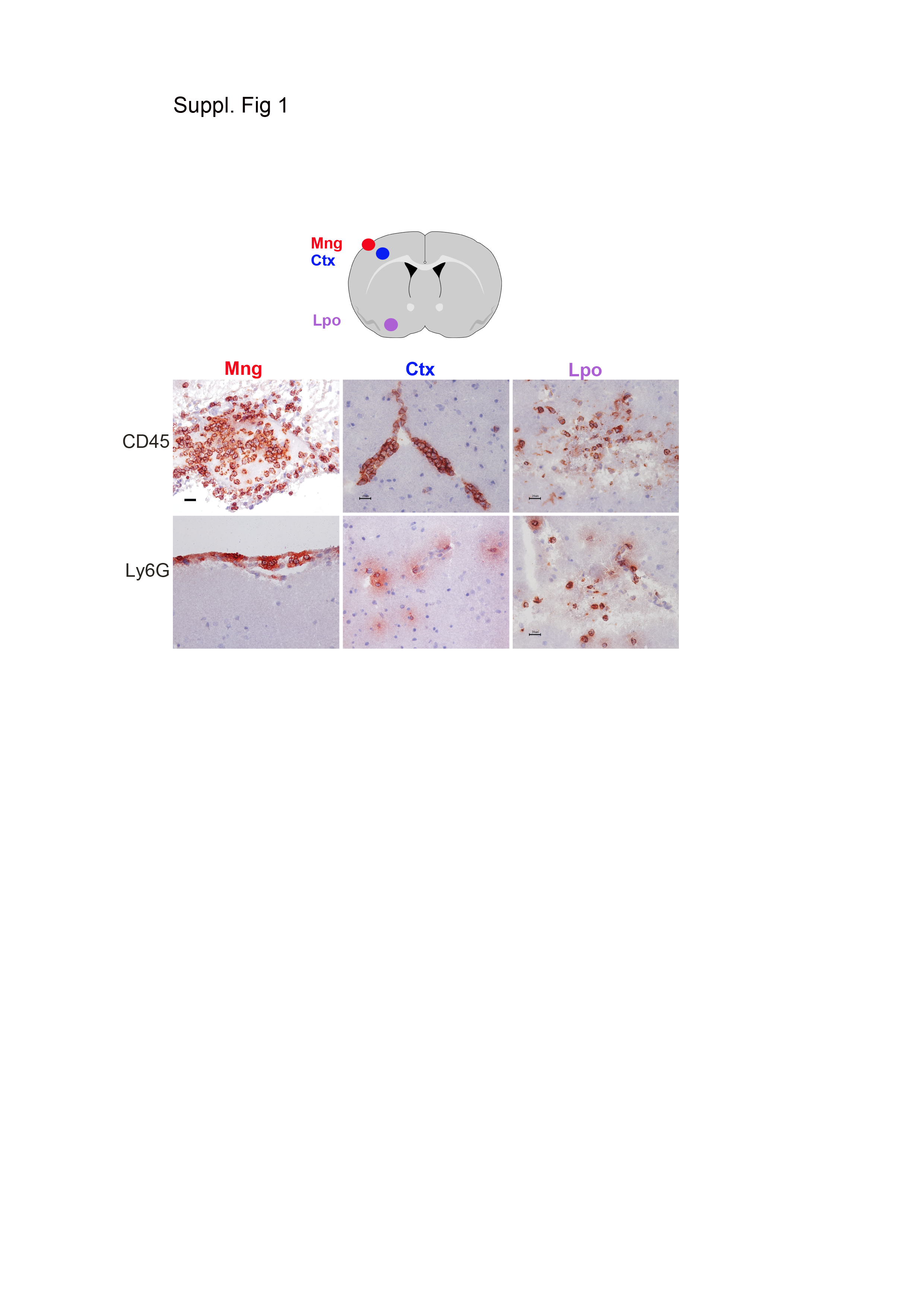

Supplement: Supplementary file 2 — Supplementary Figure 1 Moderate immune cell accumulation following 60 min tMCAO and 18h reperfusion. H&E staining of coronal CNS sections illustrating areas analysed by immunohistochemistry. Most CD45+ cells and Ly6G+ PMNs localized to the meninges (Mng), blood vessels in the cortex (Ctx) and to the lateral preoptic area (Lpo). Detection of PMNs in the lateral preoptic area (Lpo) always coincided with destruction of blood vessels and the loss of tissue integrity. Bars are 20 μm. (TIFF 25513 kb) [file 401_2012_1076_MOESM2_ESM.tif]

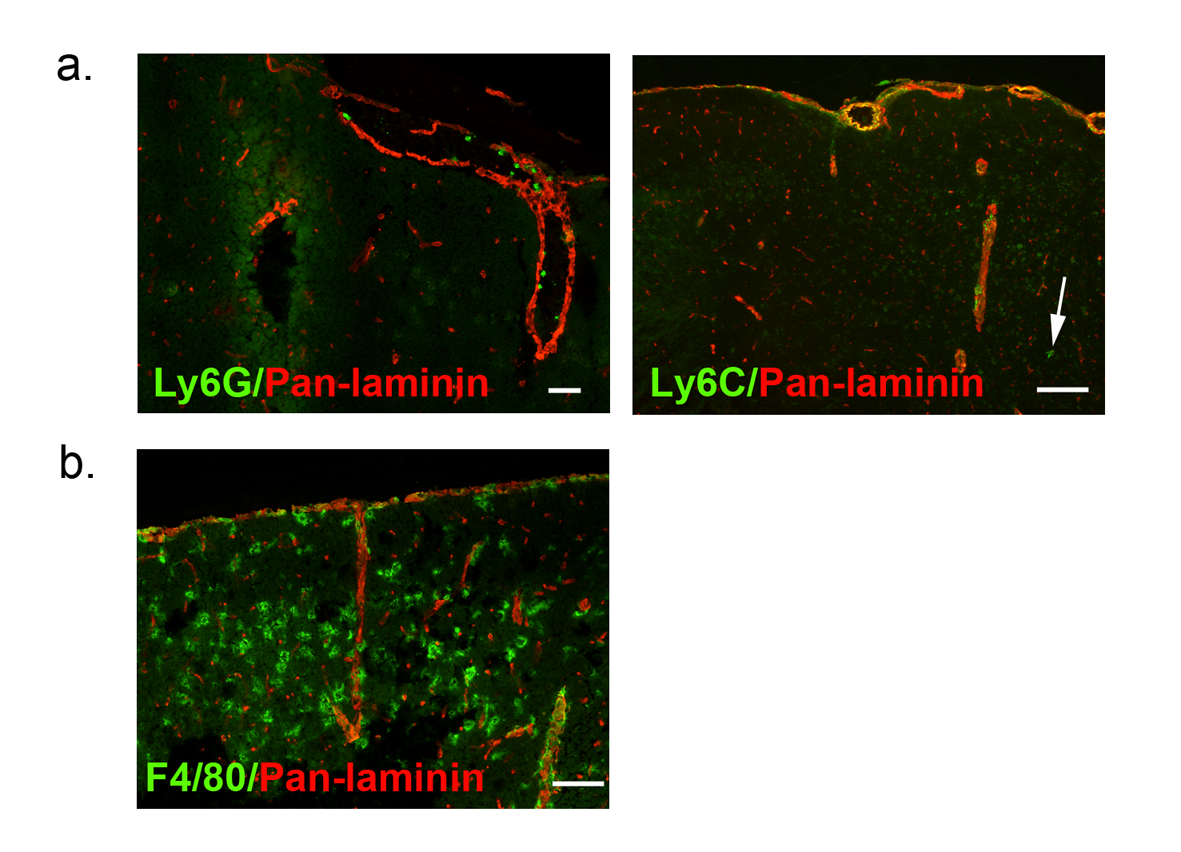

Supplement: Supplementary file 3 — Supplementary Fig 2. Immunofluorescence staining for PMNs, monocytes and macrophages in 60 min tMCAO samples. A) Immunofluorescence staining for pan-laminin to mark the border of the NVU together with Ly6G reveals that at 24h after ischemia/reperfusion PMNs localize predominantly to vessel lumina in the meninges and cortex, while at the same time point Ly6C+ monocytes although scarce occasionally appear in the brain parenchyma (arrow). b) At 72h, F4/80+ macrophages /activated microglia are abundant within and outside vessels. Bars are 70 μm. (TIFF 2977 kb) [file 401_2012_1076_MOESM3_ESM.tif]

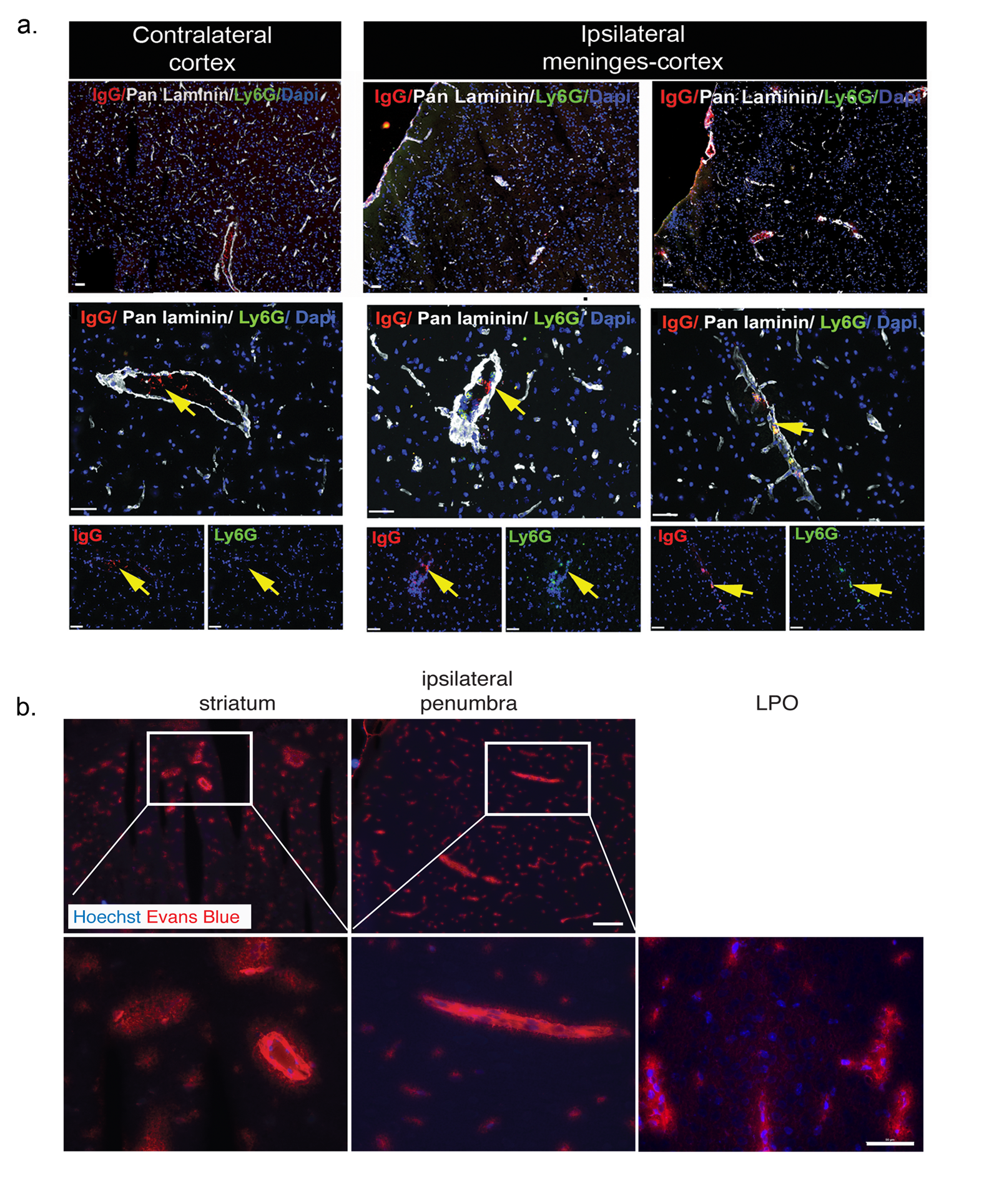

Supplement: Supplementary file 4 — Supplementary Fig 3. Ly6G + PMN accumulation does not correlate with increased vascular permeability. 60 min tMCAO at 24 h reperfusion data are shown. a) Two upper panels show contralateral and ipsilateral brain hemispheres triple stained for mouse IgG, to visualize serum protein extravasation into the CNS parenchyma, Ly6G+ PMNs and pan-laminin to mark the border of the NVU; nuclei are visualized by DAPI. Lower panels show single stainings of the vessels marked by the arrows in the panels immediately above. The arrows mark positions of IgG staining alone or in association with PMNs within vessel lumina, showing no strict correlation between Ly6G+ cells and IgG. Bar is 40 μm. b) Perfusion with Hoechst 33258 and Evans Blue revealed the integrity of the majority of the BBB microvessels in the ischemic striatum and penumbra (top row, bars are 100 μm). On rare occasions, Evans Blue penetrated dilated vessels in the ischemic hemisphere where diffuse perivascular extravasation is seen beyond the Hoechst-labeled nuclei of the endothelial cell layer; Lpo is in the lateral preoptic area (bottom row shows high magnification, bars are 50 μm). The permeability marker remained strictly confined to the vessel lumen in the contralateral hemisphere. Similar results were observed in the Dextran infused mice (not shown). (TIFF 8940 kb) [file 401_2012_1076_MOESM4_ESM.tif]

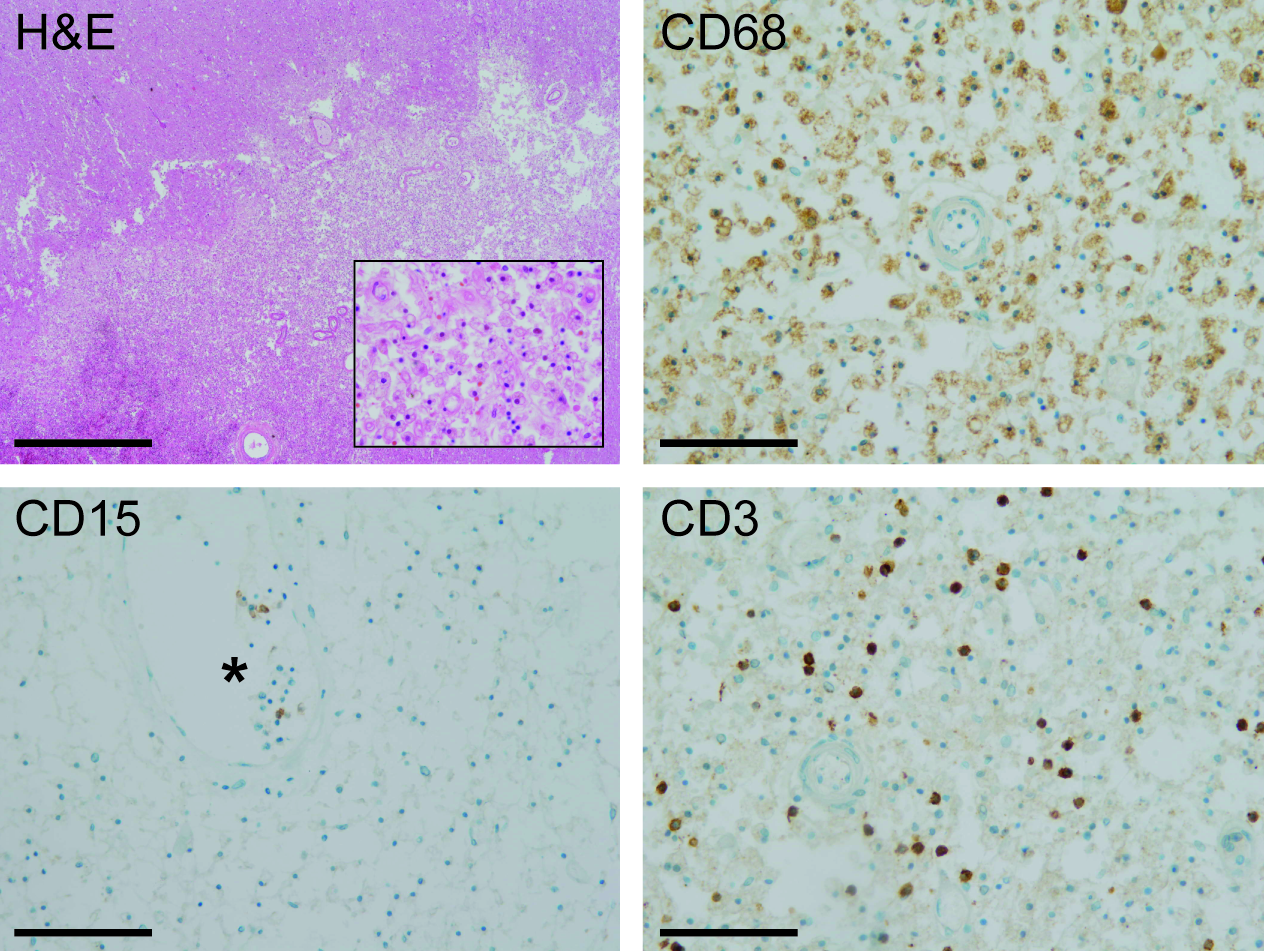

Supplement: Supplementary file 5 — Supplementary Fig 4. Histopathology of human subacute stroke specimens (Stage II). (left upper) H&E stain indicating macrophage-rich lesion (see insert for higher magnification) which is sharply delimited from the surrounding penumbra (scale bar: 1mm); (right upper) the largest cell population in subacute human stroke lesions consists of CD68-positive macrophages/microglia (scale bar: 100µm); (left, lower) CD15-positive PMNs were virtually absent in those lesions while still being present within blood vessels (asterisk; scale bar: 100µm); (right lower) a moderate number of CD3-positive T-lymphocytes is also present in this subacute human stroke lesion (scale bar: 100µm). Data shown are from a 45-year-old male patient suffering from a large ischemic infarct within the territory of the right middle cerebral artery (for details see Supplementary Table 1). (TIFF 4697 kb) [file 401_2012_1076_MOESM5_ESM.tiff]
